# Idiopathic Granulomatous Mastitis as a Benign Condition Mimicking Inflammatory Breast Cancer: Current Status, Knowledge Gaps and Rationale for the GRAMAREG Study (EUBREAST-15)

**DOI:** 10.3390/cancers16193387

**Published:** 2024-10-03

**Authors:** Natalia Krawczyk, Thorsten Kühn, Nina Ditsch, Steffi Hartmann, Oreste Davide Gentilini, Annette Lebeau, Jana de Boniface, Markus Hahn, Güldeniz Karadeniz Çakmak, Sadaf Alipour, Vesna Bjelic-Radisic, Hans-Christian Kolberg, Toralf Reimer, Maria Luisa Gasparri, Nikolas Tauber, Melissa Neubacher, Maggie Banys-Paluchowski

**Affiliations:** 1 Department of Gynecology and Obstetrics, University Hospital Duesseldorf, 40225 Duesseldorf, Germany; 2 Department of Gynecology and Obstetrics, University of Ulm, 89070 Ulm, Germany; 3 Department of Gynecology and Obstetrics, Die Filderklinik, 70794 Filderstadt, Germany; 4 Breast Cancer Center, University Hospital Augsburg, 86156 Augsburg, Germany; 5 Department of Gynecology and Obstetrics, University Hospital Rostock, 18059 Rostock, Germany; 6 Department of Breast Surgery, San Raffaele University and Research Hospital, 20132 Milan, Italy; 7 Institute of Pathology, University Medical Center Hamburg-Eppendorf, 20246 Hamburg, Germany; 8 Private Group Practice for Pathology Lübeck, 23552 Lübeck, Germany; 9 Department of Medical Epidemiology and Biostatistics, Karolinska Institutet, 17176 Stockholm, Sweden; 10 Department of Surgery, Breast Unit, Capio St Göran’s Hospital, 11281 Stockholm, Sweden; 11 Department for Women’s Health, University of Tübingen, 72076 Tübingen, Germany; 12 Breast and Endocrine Unit, General Surgery Department, Zonguldak BEUN The School of Medicine, Zonguldak 67600, Türkiye; 13 Breast Diseases Research Center, Cancer Institute, School of Medicine, Tehran University of Medical Sciences, Tehran 1419733141, Iran; 14 Department of Surgery, Arash Women’s Hospital, School of Medicine, Tehran University of Medical Sciences, Tehran 1653915911, Iran; 15 The Breast Unit, Helios University Hospital Wuppertal, University Witten-Herdecke, 42283 Wuppertal, Germany; 16 Department for Gynecology and Obstetrics, Marienhospital Bottrop, 46236 Bottrop, Germany; 17 Department of Gynecology and Obstetrics, Ospedale Regionale di Lugano EOC, 6900 Lugano, Switzerland; 18 Centro di Senologia della Svizzera Italiana (CSSI), Ente Ospedaliero Cantonale, via Pietro Capelli 1, 6900 Lugano, Switzerland; 19 Faculty of Biomedical Sciences, Università della Svizzera Italiana (USI), via Giuseppe Buffi 13, 6900 Lugano, Switzerland; 20 Department of Gynecology and Obstetrics, University Hospital Schleswig-Holstein Campus Lübeck, 23538 Lübeck, Germany

**Keywords:** idiopathic granulomatous mastitis, breast inflammation, steroids, methotrexate, immunosuppressants, surgery

## Abstract

**Simple Summary:**

Idiopathic granulomatous mastitis (IGM) is a rare breast disease that can be mistaken for inflammatory breast cancer. It requires a tissue biopsy for an accurate diagnosis. Even though it is not cancerous, IGM can cause emotional distress because of severe pain and ensuing breast deformity. It is important to distinguish IGM from other breast inflammations caused by infections. IGM is mostly found in premenopausal women, often after pregnancy or breastfeeding. Smoking and contraceptive use might be risk factors, but the evidence is unclear. The treatment options include NSAIDs, steroids, immunosuppressants, surgery, prolactin suppressants, and antibiotics; many patients relapse, making treatment challenging. This review summarizes current information, which mostly comes from case reports and small studies, and presents GRAMAREG as a registry for IGM initiated by the EUBREAST Study Group, which aims to collect detailed data on IGM to improve the knowledge base for diagnosis and treatment.

**Abstract:**

Background: Idiopathic granulomatous mastitis (IGM) is a rare, benign inflammatory breast condition often mistaken for inflammatory breast cancer and, therefore, requires a biopsy for accurate diagnosis. Although not cancerous, IGM can cause emotional distress because of severe pain and ensuing breast deformity. Differentiating IGM from other breast inflammations caused by infections is essential. IGM mostly affects premenopausal women and is potentially associated with recent pregnancies and breastfeeding. The risk factors, including smoking and contraceptive use, have inconsistent associations. Steroid responses suggest an autoimmune component, though specific markers are lacking. Methods: We performed a narrative review on potential risk factors, diagnostics, and therapy of IGM. Results: Diagnostics and clinical management of IGM are challenging. The treatment options include NSAIDs, steroids, surgery, antibiotics, immunosuppressants, prolactin suppressants, and observation, each with varying effectiveness and side effects. Conclusions: Current IGM treatment evidence is limited, based on case reports and small series. There is no consensus on the optimal management strategy for this disease. The GRAMAREG study by the EUBREAST Study Group aims to collect comprehensive data on IGM to improve diagnostic and treatment guidelines. By enrolling patients with confirmed IGM, the study seeks to develop evidence-based recommendations, enhancing patient care and understanding of this condition.

## 1. Introduction

Idiopathic granulomatous mastitis (IGM), also known as lobular granulomatous lobular mastitis, is a rare inflammatory breast disease of uncertain etiology, first described by Kessler and Wolloch in 1972 [1]. Even though it is a benign condition, it can mimic breast cancer, requiring a histological evaluation to exclude malignancy and to confirm the IGM diagnosis. The local symptoms of IGM are mostly severe, lasting up to many weeks or months, potentially causing severe pain and a high level of emotional distress.

It is worth noting that IGM needs to be differentiated from granulomatous inflammation of the breast that can appear in duct ectasia, as a reaction to foreign material, or in association with specific infections (e.g., mycobacteria, fungi, parasites) as well as in the course of systemic granulomatous diseases such as sarcoidosis. Further, cystic neutrophilic granulomatous mastitis (CNGM) was recently described as a specific form of granulomatous inflammation of the breast linked to infection with *Corynebacterium* species. However, it has not been fully clarified whether CNGM represents a distinct entity or, rather, a subtype of IGM [2].

Due to the rarity of the disease and the significant degree of suffering in affected patients, the European Breast Cancer Research Association of Surgical Trialists (EUBREAST) has initiated the GRAMAREG (GRAnulomatous MAstitis REGistry) study. GRAMAREG is a multinational cohort study with a combined retrospective/prospective design, which enrolls patients with histologically confirmed IGM. This study aims to evaluate risk factors, clinical symptoms, imaging appearance, therapeutic management as well as patient-reported outcomes in this uncommon disease.

The registry is designed to yield high-quality evidence for both patients and clinicians, potentially allowing evidence-based adoption of recommendations regarding diagnostics and treatment into national and international guidelines. This narrative review aims to identify the current knowledge regarding IGM to help inform the GRAMAREG study design.

## 2. Current Knowledge of IGM

### 2.1. Prevalence

The current body of evidence is limited to case reports and usually retrospective case series. Therefore, valid data on the prevalence of this condition are lacking. A report by the Centers for Disease Control from 2008 showed a prevalence of IGM of 2.4 per 100,000 Hispanic women in Indiana aged 20–40 years [3]. Epidemiological reports from Europe or Asia do not exist. However, since most published IGM cases were reported from the Middle East and Asia, the incidence appears to be higher in these regions than in Western Europe [4,5,6,7]. Interestingly, also in case series reported from Europe or the U.S., IGM patients are frequently of non-Caucasian ethnicity even though there is no established predisposition in one specific ethnic group [3,8,9,10,11].

### 2.2. Risk Factors and Etiology

Several etiological factors for IGM, including pregnancy and lactation, hyperprolactinemia, smoking, oral contraceptive use, autoimmune disorder, and infection, have been discussed in the literature. Importantly, no association between IGM and breast cancer has been found to date, so patients can be reassured that the condition is not associated with an increased risk for malignancy.

Based on published studies, IGM is considered to predominantly occur in premenopausal women with a mean age between 32 and 36 years [4,5,7,9,10,11,12,13]. However, IGM has been rarely described in adolescent females [14] and in male patients [15]. Some of the male cases were associated with exposure to a high level of estrogen, which is comprehensible since IGM is localized in breast lobules, and the male breast typically differentiates these structures only in cases of hyperestrogenism [15,16,17].

The vast majority of female patients have a history of childbearing and lactation [4,5,7,8,10,11,18], often with IGM diagnosis reported within 5 years after the latest pregnancy [8,9,10,12]. In patients without a gestational history, IGM might be associated with high levels of prolactin [19,20], suggesting a potential role of hyperprolactinemia in its pathogenesis [21]. In most of the published studies on IGM, however, prolactin levels were not evaluated, and only a minority of published cases have been linked to hyperprolactinemia, e.g., due to antipsychotic medication or intracranial tumors such as pituitary adenomas [21].

While smoking represents a major risk factor for periductal mastitis [22], the association between smoking and IGM is much less clear. Depending on the published series, varying smoking rates were reported among IGM patients. In a study by Baslaim et al., none of 20 IGM patients had a history of smoking [23], whereas 172 of 720 patients (24%) in the large series by Uysal et al. [24], 16 of 46 (35%) according to Oran et al. [25], and 14 of 18 patients (78%) in the series by Asoglu et al. [26] were smoking at the time of or prior to IGM diagnosis. In the analysis by Uysal et al., smoking was significantly associated with an elevated risk of recurrence of IGM. However, since smoking habits have not been reported in several IGM studies, only limited data are available on this subject [4,5].

Another risk factor of IGM discussed in the literature is the use of oral contraceptives. While some case studies report rates of contraceptive intake ranging from 13 to 42% among IGM patients [5,24,25,27], others report that none of the analyzed IGM patients had a history of contraceptive use [4,23,28], suggesting that there is no conclusive association between oral contraceptives and the incidence of IGM.

Since several groups reported a good response to steroid or immunosuppressive therapy in IGM patients, an autoimmune etiology of this disease has been discussed. However, neither an autoimmunological mechanism nor specific serological markers for IGM have been established to date. In a small case series by Ozel et al., six out of eight surgically treated patients presented positive for rheumatoid factor (RF) and two for antinuclear antibody (ANA) and anti-double stranded DNA antibody (anti-dsDNA). Three of those patients had a disease recurrence and responded well to the steroid treatment in the recurrence setting [28]. Single-case reports and small case series describing IGM in association with erythema nodosum [29,30] and other rheumatic diseases [14,31,32] exist in the literature, suggesting a systematic autoimmune reaction in the context of this condition. However, in a series of 18 IGM cases by Asoglu et al., all patients were negative for RF and ANA [26]. Similarly, in the analysis by Altintoprak et al. in 26 IGM patients, only two showed 1:100 ANA levels, regarded as positive [33]. In the majority of published cases, autoimmune markers were not evaluated, and systemic symptoms suggesting autoimmune reactions were not described.

Increasing evidence about the role of corynebacterium species in the etiology of granulomatous mastitis (GM) has been gathered in the last decade, with C. kroppenstedtii being the most commonly identified pathogen [34,35,36,37,38,39,40]. Corynebacteria are Gram-positive and belong to the skin flora [41]. Corynebacterium species have been detected on Gram stain, in bacterial culture, or by 16S rRNA gene sequencing of the microbiological, histological, or cytological specimens in several cases of GM [34,35,36,37,38,39,40,42]. Due to the specific histopathological pattern with (1) small cystic spaces within the granulomas (also called lipogranulomas), which are (2) sometimes filled with Gram-positive bacteria, the term cystic neutrophilic granulomatous mastitis (CNGM) has been introduced in 2011 by Renshaw et al. [2] (see Table 1). Since the histopathological characteristics of CNGM and IGM overlap, some authors consider CNGM a subtype of IGM, while others suggest that CNGM represents a distinct entity of GM [2,43]. Since IGM is, by definition, a noninfectious disease with the absence of microorganisms as a typical feature, CNGM should rather be considered a distinct entity of GM, especially when Gram-positive bacteria have been identified within the lipogranulomas. Interestingly, CNGM can be diagnosed based on its distinct histopathological criteria even when no corynebacteria have been detected [43], and CNGM cases with a mixed bacterial population as causative pathogens have also been reported in the literature [44]. Furthermore, several authors suggest that the detection of these bacteria, as well as the differentiation between colonization, contamination, and infection in those cases where corynebacterium species were detected, can be difficult [42]. Therefore, it remains unclear whether some cases previously reported as IGM, in fact, may represent examples of CNGM, in which the specific cystic spaces and Gram-positive bacteria were not identified or recognized [43].

### 2.3. Histopathological Features

A pathologic hallmark of IGM is primarily lobulocentric granulomas that often contain neutrophils. Depending on the amount of neutrophils, necrotic foci (microabscesses) may be present within the granuloma. However, in contrast to tuberculosis, true caseous necrosis (noncaseating granulomas) is not seen [43], even though breast tuberculosis can be histologically mistaken for IGM in rare cases [45]. IGM should also be distinguished from sarcoidosis, characterized by noncaseating granulomas with giant cells in the inter- or intralobular stroma, although the incidence of mammary sarcoidosis, especially as a singular manifestation, is very low [43,46]. As mentioned above, differentiation between IGM and CNGM can be difficult since both conditions share some histopathological features. The discrimination between IGM and CNGM is based on (1) the presence of small cystic spaces surrounded by neutrophils within the granulomas, thought to be caused by dissolved lipid (lipogranulomas), and (2) the identification of Gram-positive bacteria (mostly Corynebacterium species) within these cystic spaces in some cases [43]. Further, granulomatous inflammation of the breast may be caused by other infections (such as mycobacteria, fungi, and parasites), duct ectasia, or foreign body reactions. Histological features of granulomatous breast lesions are summarized in Table 1.

### 2.4. Clinical Symptoms and Diagnostic Workflow

**Figure 1:**
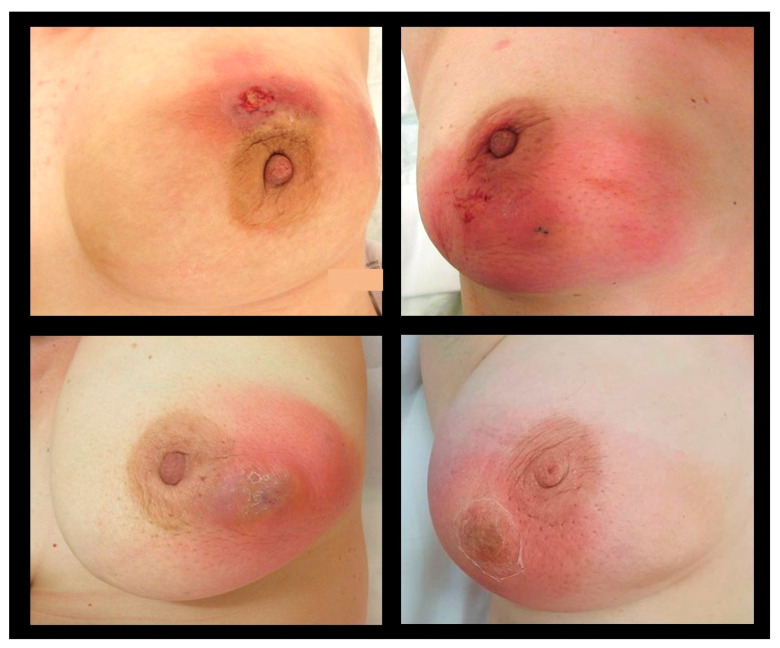
Typical clinical presentation in patients diagnosed with IGM.

The local symptoms of IGM are often severe, lasting up to many weeks or months, causing significant pain and sometimes mimicking inflammatory breast cancer (Figure 1). The main clinical symptom reported in published studies is a palpable, often large, painful mass with overlying erythema [4,7,9,25,27,47] (Table 2). Some patients present initially with an abscess [4,6,7,15,25], skin ulceration, or sinus formation [5,15]. Nipple retraction or nipple discharge can occur depending on the localization of the mass [5,15,27,47]. Axillary lymphadenopathy is described in up to 32% of IGM patients [4,15,25,27,47]. In the majority of IGM patients, symptoms present unilaterally, while bilateral IGM is reported in 1–8% of published cases [4,5,11,25,48,49]. Few studies report extramammary symptoms in IGM patients, such as arthralgia and erythema nodosum [5,47]. Imaging findings without any clinical symptoms, where a biopsy was performed to rule out malignancy, have also been reported [11].

Since IGM symptoms often mimic breast cancer, including inflammatory breast cancer, IGM should be confirmed by histology to investigate its microscopic features and to exclude malignancy and infectious and inflammatory disorders of the breast. The method of choice for histological assessment is a core needle biopsy (CNB); several reported IGM cases, however, were diagnosed by a surgical biopsy or fine needle aspiration [6,9,27,47] (Table 2).

In this context, we should keep in mind that IGM is an exclusion diagnosis and secondary causes of granulomatous inflammation of the breast must be ruled out (Table 1). Exclusion workflow mostly implies microbiological staining and culture (from wound/abscess swab or biopsy material) and special histological tissue staining to exclude specific infections, such as tuberculosis or other infections causing granulomas, such as fungal or other acid-fast bacilli infections (Table 1). Several authors, especially from tuberculosis-endemic countries, include chest radiography and tuberculin skin tests and/or tuberculosis polymerase chain reaction tests into the IGM exclusion workflow [9,25,47]. Depending on chest radiography findings, the serum angiotensin-converting enzyme (ACE) level can be measured to exclude sarcoidosis, which can be best ruled out histologically (see histological features) [43,47].

The clinical course of the disease is usually chronic. Patients commonly develop recurrent breast abscesses, which do not respond to antibiotics and require repeat incisions or drainage [47]. The recurrence rate in published studies is up to 25% [5,7,11,24,25,27]. The degree of suffering of IGM patients is, therefore, high. However, due to the rarity of the condition, the existing evidence for IGM and its clinical course is still limited.

### 2.5. Presentation on Imaging

Radiologic features of IGM are not specific and often overlap with those of malignant lesions. The most common imaging modality performed in IGM patients is ultrasound, followed by mammography and magnetic resonance imaging (MRI) [4,24,25].

**Figure 2:**
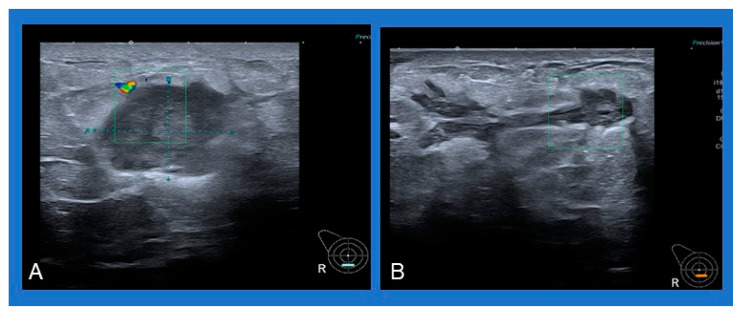
Typical sonographic appearance of IGM: (**A**) heterogeneous mass with ill-defined margins, (**B**) multiple fluid collections with floating debris.

A common sonographic appearance described in several studies is an irregular hypoechoic or heterogeneous mass with ill-defined margins [4,6,50]. Single or multiple fluid collections with floating debris are also often reported [4,6,50] (Figure 2). A typical mammographic finding is an irregular mass, followed by focal asymmetry, usually without microcalcifications [4,50].

Studies on MRI in IGM patients report a large variability of findings in small populations. Most commonly reported MRI changes are heterogeneously enhancing T2-hyperintense lesions, rim-enhancing masses with regional non-mass enhancement (NME), or NME without accompanying mass [51].

In conclusion, based on current evidence, radiologic imaging cannot be used to distinguish between an IGM and a malignant lesion [52]. Moreover, most studies do not report imaging findings at all [5,7,11], and only a few report any assigned BIRADS scores (mostly BIRADS 3 or BIRADS 4, rarely BIRADS 5) [4,50,53,54]. Since mammography can cause severe pain in cases of extensive swelling or abscesses, ultrasound should be considered the first-line examination in IGM patients, followed by mammography if appropriate and MRI in selected cases.

### 2.6. Therapy

Various treatment strategies, including an observational approach, antibiotics, high-dose steroids, immunosuppressive agents, bromocriptine, and surgical resection or even mastectomy, have been reported [9,12,55,56]. No evidence-based treatment recommendations for IGM are available to date.

### 2.7. Observation

Since the spontaneous resolution of IGM has been reported in the literature, some authors consider IGM as a self-limiting condition. In a study by Lai et al., a spontaneous resolution of symptoms was described in four out of eight IGM patients with a time interval until complete resolution of up to 2 years [57]. In the analysis by Azizi et al. on 474 IGM patients, 15.1% did not receive any therapy, and IGM symptoms resolved spontaneously within 9 months [5]. However, considering severe clinical symptoms reported in the majority of IGM patients, an observational approach seems to be not justified for a prolonged time period.

### 2.8. NSAIDs

Since IGM patients often present with pain and/or signs of inflammation, NSAIDs play an important role as an analgetic and/or anti-inflammatory agent in the therapy of this condition. In the trial by Bhattarai et al., 29 of 63 IGM patients (46%) received NSAIDs as adjuncts to antibiotics or steroids with variable results [54]. Studies that have analyzed NSAIDs as independent agents for IGM treatment are rare. In the small case series by Freemann et al., one IGM patient was treated with NSAIDs alone; however, her outcome was not reported [9]. In the retrospective study by Kaviani et al. on 374 IGM patients, 41.8% were treated with NSAIDs for several weeks (17.5+/−14.2), and 31.5% recovered completely after this treatment [58]. Due to the long therapy duration, potential side effects on the gastrointestinal tract, renal system, and other organs, especially if NSAIDs are being administrated in combination with antibiotics or steroids, should be taken into account. These can be potentially minimized by rational prescribing and by the administration of mucosal protective agents as co-therapy.

### 2.9. Antibiotics

The majority of patients presenting with IGM are initially treated empirically with antibiotics for presumed bacterial mastitis [6,7,9,10,11,55]. However, given no improvement and negative microbiological cultures, antibacterial medication is commonly discontinued after a short course of therapy [9,11]. Some studies report a secondary bacterial infection or coinfection and suggest a continuation of antibiotics even after the IGM diagnosis has been confirmed histologically [6,25].

In case corynebacterium species have been detected, mostly in combination with specific histomorphology characterized by lipogranulomas, a diagnosis of CNGM is made. These patients should be treated with targeted antibiotics. Several medications, including rifampicin, clarithromycin, clindamycin, or trimethoprim-sulfamethoxazole, come into consideration because of the lipophilic properties of these agents and their high ability to penetrate breast tissue. In contrast, ß-lactam antibiotics or fluoroquinolones are expected to be less effective because of their low lipid solubility [40]. Antibiotic treatment for CNGM can be required for several weeks [59].

### 2.10. Steroids

The first-line medical treatment after histological confirmation of IGM is systemic steroids, particularly prednisolone or prednisone, first proposed by DeHertogh et al. in 1980 [60]. Since then, several studies have demonstrated a high efficacy of these substances in IGM patients. In an observational prospective analysis by Pandey et al., including 44 IGM patients treated with steroids alone, an initial response rate of 100% and a complete resolution rate of 80% were reported [11]. However, 23% of patients suffered a subsequent recurrence during the follow-up of 6–12 months, even after the initial complete resolution of symptoms. Recurrences were treated successfully with a second course of steroids in all patients [11]. Other, mostly retrospective studies described similar effects of steroid monotherapy with response rates up to 93% (Table 3). However, several published IGM case series reported no detailed information regarding the final patient outcome after steroid treatment (complete resolution vs. partial resolution vs. no response) [5,24,49]. Moreover, studies on IGM patients treated with steroids rarely specify the administered regimen [9,10,24]. In their small prospective randomized trial, Montazer et al. compared a low dose (5 mg daily for 2 months) vs. a high dose (50 mg, 25 mg, and 12.5 mg for three days, respectively, and 5 mg daily afterward for 2 months) prednisolone regimen in 30 IGM patients [61]. A high-dose therapy showed a higher remission rate compared with the low-dose regimen (93% vs. 53%, respectively, *p* = 0.03) and lower recurrence rates among patients with remission (0 vs. 37.5%, respectively, *p* = 0.04). A commonly used daily dose of systemic prednisolone as a monotherapy for IGM is 0.5–1 mg/kg [27,47,62], even though there is no particular recommended standard regimen to date. Since the Cushing threshold for prednisolone or prednisone is considered at 7.5 mg per day, typical side effects can occur, especially because of the long treatment duration and the fact that the medication needs to be tapered over time before being discontinued. Some examples of steroid treatment regimens in published studies on IGM patients are presented in Table 3.

Apart from systemic therapy, topical steroids, and local steroid injections have been used in small studies on IGM patients in order to reduce systemic side effects. Altintoprak et al. evaluated topical prednisolone (0.125% twice a day on alternate days for 4 days, with a subsequent interval of 3 days; 1-week cycle) in 28 IGM patients with skin changes (ulceration, fistula, inflammation). After a mean treatment duration of 8.2 weeks (range 4–12 weeks), a complete resolution of local symptoms was reported in all patients [64]. During an average follow-up of 37.2 months, 10.7% of patients (3/28) experienced a relapse and were treated with the same regimen again. Two of these patients showed a long-term response to repeat treatment.

Recently, Toktas et al. compared a local steroid therapy with systemic steroid treatment in a prospective study on 78 IGM patients. Forty-six patients received an intralesional injection of 20 mg/1 cm^3^ triamcinolone acetonide once a month in combination with topical administration of triamcinolone acetonide 0.1% pomade on the affected skin region twice a day for 1 month and 26 patients received systemic therapy with 32 mg methylprednisolone per day for 1 month. Patients from both groups were evaluated monthly, and treatment was repeated in patients without a complete response. After the third course of treatment, response rates (complete or partial response) were significantly higher, and recurrence rates were significantly lower in the local therapy group compared with the systemic therapy group (93.5% vs. 71.9%, *p* = 0.012 and 8.7% vs. 46.9%, *p* = 0.001, respectively). Patients from the local therapy group also had fewer systemic side effects [65]. In a study by Cabioglou et al., local steroids were compared with the combination of local (topic + intralesional) and systemic low-dose steroid treatment in 51 IGM patients, showing similar response rates in both treatment groups with less side effects in patients treated with local steroids only [66]. Both research groups suggest that local steroid therapy should be considered as a new first-line treatment for IGM patients.

### 2.11. Methotrexate

Methotrexate (MTX), a folic acid antagonist, is a cytostatic agent that can be used in IGM, often as a second-line treatment. In the majority of published cases, MTX was used in patients who had not responded to steroids or who suffered from systemic steroid side effects. It can be administrated as a monotherapy or in combination with steroids in order to reduce their dose [4,9,47,67,68]. The common MTX dose also used to treat other inflammatory disorders is 7.5–10 mg per week +/− prednisolone or prednisone 10–15 mg per day [4,47]. In case of insufficient response, the MTX dose can be increased up to 20 mg per week [68]. In one of the largest IGM series by Aghajanzadeh et al., including 206 cases, 56 patients who had not responded to steroids alone were treated with a combination of MTX and steroids with a response rate of 71% [4]. Papila et al. treated 64 IGM patients, 56 of whom were steroid-resistant, with MTX monotherapy 15 mg/week for 24 weeks, with complete resolution achieved in 52 (81%). Nausea was the only side effect of MTX reported in three patients in this study [68]. In a small series by Kafadar et al., a low-dose MTX regimen (5 mg per week) was combined with low-dose prednisone (8 mg per day) as a second-line treatment in 17 IGM patients. After a treatment duration of 2–3 months, a complete resolution was observed in 10 patients (58.5%), partial resolution in 3 (17.6%), and no response in 4 patients (23.5%). No side effects of MTX were reported. In patients with no or partial response, a surgical excision of the lesion was performed [69]. Haddad et al. reported excellent responses to MTX alone or in combination with low-dose steroids as a first-line treatment in 13 IGM patients. All patients showed a complete resolution of symptoms and a recurrence rate of 17.6% during a follow-up of 16.2 months. One patient suffered hair loss, whereas other reported side effects were mild (nausea, mild headache, decreased appetite, and a mild increase in liver function tests) [67]. In a recently published study by Shojeaian et al. on a big cohort of 318 recurrent IGM patients, 94.3% achieved complete response after treatment with a combination of MTX (12.5 mg/m^2^/week) and low-dose steroids (12.5 mg/m^2^/day) up to 18 months [70].

In conclusion, MTX may be considered as an alternative in steroid-resistant IGM patients or as a steroid-sparing agent in first- or second-line treatment in selected cases showing similar response rates to steroid monotherapy. However, the fact that MTX is a cytostatic agent with known teratogenic effects needs to be taken into account, particularly because most IGM patients are women of reproductive age. Moreover, a supplementation of 5–10 mg folic acid weekly should be recommended [68].

### 2.12. Other Immunosuppressants

Apart from steroids and MTX, further immunosuppressants have been studied in IGM patients to date. Few small series reports on the use of azathioprine as a steroid-sparing agent with limited side effects [71,72]. Recently, a case report on a pregnant IGM patient successfully treated with long-term therapy with azathioprine and allopurinol was published, considering this agent as an immunosuppressant that can be administrated during pregnancy and lactation [73]. Further immunosuppressive substances such as colchicine or hydroxychloroquine were also administrated in IGM either as steroid-sparing agents to control a mildly active disease or as a maintenance therapy to avoid disease exacerbation [74]. Recently, a case report on successful topical treatment with the immunomodulator agent imiquimod in 2 patients with refractory IGM was published [75].

### 2.13. Prolactin Suppressants

Since hyperprolactinemia is a possible etiological factor in IGM, prolactin-suppressing medications are discussed as potential therapeutic agents. In a study by Aghajanzadeh et al. on 206 IGM patients, 16 patients resistant to steroid and MTX therapy were treated with a combination of steroids and bromocriptine 5–10 mg per day. Five of these patients (31%) showed good response to this regimen, suggesting that adding bromocriptine may show favorable effects in selected patients with IGM [4].

### 2.14. Surgery

Before systemic steroid therapy became widely established, surgical excision was a mainstay of treatment in IGM patients with an aim to remove the affected area completely. Since the IGM symptoms are often extensive or multifocal, the required surgical procedure for complete excision was mostly a wide excision, quadrantectomy, or even a mastectomy [12,76,77]. Apart from excisional procedures, patients who initially present with an abscess or develop an abscess in the course of the disease are often treated with incision and drainage.

After systemic steroids became widely used as a first-line medical treatment of IGM, a de-escalation of surgical therapy became possible, and surgery can nowadays be completely avoided in many patients. A combination of steroids and surgery was studied in several IGM case series, showing low recurrence rates [24,27]. In their meta-analysis of 15 IGM series, Lei et al. showed better outcomes after combined treatment with steroids and surgery (i.e., excision and drainage) compared with surgery alone or steroid therapy alone [78]. However, Zhou et al. found no statistical differences in recurrence rates after medical (medical alone or medical + surgical) vs. only surgical IGM treatment in their analysis of 10 retrospective series on 1101 IGM patients [79]. On the other hand, a meta-analysis of 21 studies on 970 IGM patients by Ma et al. showed the best outcomes after surgical management, followed by a combination of steroids and surgery and steroids single therapy [80].

In conclusion, it remains to be clarified whether surgery alone, surgery in combination with medical therapy, or no surgery at all represents the best approach for IGM patients. All available data arise from mostly small, retrospective studies and are very heterogeneous. The interpretation of these data remains particularly challenging because some authors differentiated between a combination and monotherapy approaches while others did not. Moreover, in some studies, only excisional procedures are interpreted as surgical, whereas in others, patients receiving incision and drainage for abscess management were also considered surgically treated [78,79].

## 3. GRAMAREG Study

GRAMAREG is a non-interventional multicohort study with a combined retrospective/prospective design initiated by the EUBREAST study group (http://eubreast.org/gramareg, accessed on 30 May 2024). Patients with histologically confirmed idiopathic granulomatous mastitis can be enrolled. The aim of the study is to systematically evaluate risk factors and clinical symptoms, treatment management, and patient outcomes (such as duration of symptoms and recurrence rate) of this uncommon benign disease; because of the rarity of the condition, this study is designed as an open registry with unlimited target accrual.

### 3.1. Retrospective Data Collection Phase

Patients with histologically confirmed IGM treated between 1 January 2015 and the activation of the study site can be registered. No patient-identifying information will be disclosed or transferred. Data collected during the retrospective phase of the study will be de-identified. The retrospective data collection phase does not include a prospective follow-up.

### 3.2. Prospective Data Collection Phase

**Figure 3:**
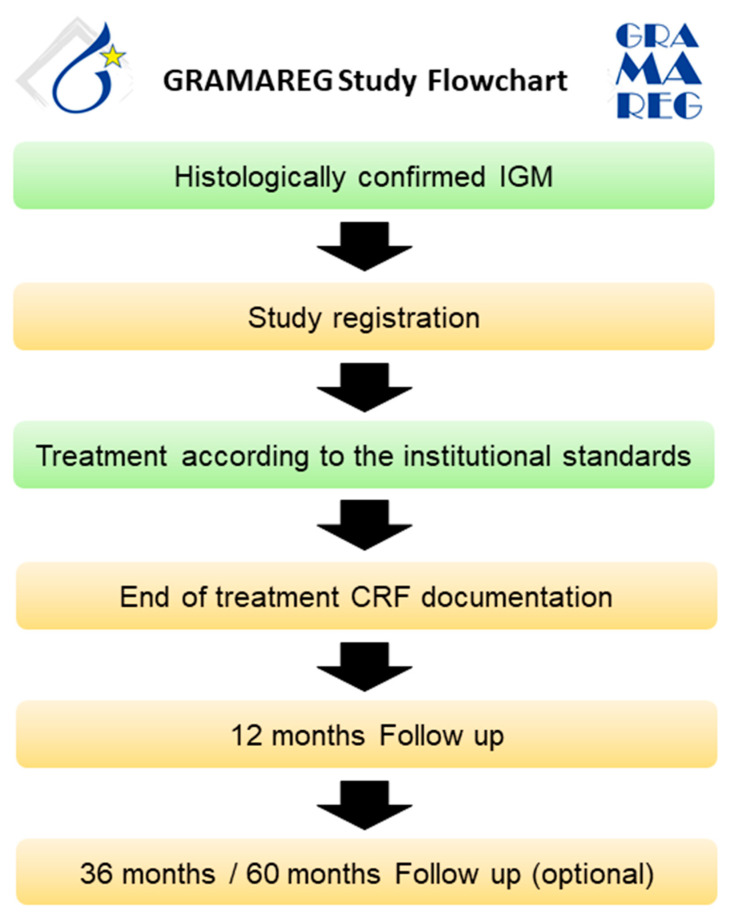
Study flowchart for the prospective data collection phase.

All patients with histologically confirmed IGM presenting at a study site after activation will be informed about their possible participation in the GRAMAREG registry. The inclusion and exclusion criteria are verified by the investigator, and written informed consent is obtained from the patient. Diagnostic management and treatment should be conducted according to institutional standards. Since the GRAMAREG study is a non-interventional trial, the study sites do not deviate from their own institutional protocol at any time. The follow-up on patient status is conducted 1, 3, and 5 years after the first diagnosis (Figure 3). Table 4 demonstrates the inclusion and exclusion criteria of the GRAMAREG study.

**Primary Study Endpoints:**
  - Proportion of patients presenting with specific symptoms (e.g., pain, redness, palpable mass)
  - Duration of symptoms depending on treatment strategy

**Secondary Study Endpoints:**
  - Type and duration of systemic treatment
  - Number of surgeries, if performed
  - Recurrence rate
  - Risk factors for recurrence
  - Time between first occurring symptoms and first histological confirmation
  - Presentation on breast imaging (mammography, sonography, MRI, if performed)

## 4. Conclusions

Idiopathic granulomatous mastitis is a rare benign breast disease. Although there is no association with increased breast cancer risk, the condition may be mistaken for inflammatory breast cancer due to overlapping symptoms. Patients usually report a high level of emotional distress. Treatment strategies include surgical approaches, such as excision or drainage, NSAIDs, steroids, immunosuppressants, and prolactin-suppressing agents. However, evidence-based therapy guidelines do not exist, and different regimens are in use. To clarify the optimal diagnostic and therapeutic approaches, the EUBREAST Study Group initiated the GRAMAREG study. Hopefully, this study will provide evidence to inform future guidelines.

## Figures and Tables

**Table 1:** Key histological characteristics of granulomatous lesion of the breast (modified from [43]).

Granulomatous Lesion/Condition	Key Histological Characteristics
Tuberculosis	Necrotizing (caseating) granulomas composed of epithelioid histiocytes surrounding a central necrotic zone
Other mycobacterial, fungal, and parasitic infections	Granulomas similar to those seen in comparable infections in other sites may be necrotizing or non-necrotizing
Sarcoidosis	Non-necrotizing (non-caseating) granulomas in inter- and intralobular stroma
Mammary duct ectasia	Periductal granulomas; may have xanthogranulomatous features
Reaction to foreign body	Foreign body-type granulomas; foreign body-type giant cells; foreign material
Cystic neutrophilic granulomatous mastitis	Lobulocentric granulomas often contain cystic spaces surrounded by neutrophils; some cystic spaces contain Gram-positive bacteria
Idiopathic granulomatous mastitis	Non-necrotizing lobulocentric granulomas often contain neutrophils

**Table 2:** Clinical presentation, treatment strategy, and recurrence rates in studies on IGM patients.

Study	Patient Number	Study Origin/Patient Ethnicity	Exclusion Workflow for IGM	Clinical Symptoms N (%)	Biopsy Method n (%)	Primary Treatment Management n (%)/Resolution Rates if Reported	Median Follow-Up in Months (Range)	Relapse n (%)
Gurleyik et al., 2012 [27], retrospective, monocentric	19	Turkey	Microbiological staining (Gram, PAS, Ziehl-Neelsen)	Palpable mass 15 (79) Painful swelling 6 (31.6) Abscess 3 (15.8) Ulcers 3 (15.8) Sinus formation 3 (15.8) Axillary LAP 2 (10.5) Nipple retraction 1 (5.3)	FNA/CNB 16 (84) Excision 3 (16)	Surgical excision + steroids 19 (100)	20 (6–75)	
Oran et al., 2013 [25], retrospective, multicentric	46	Turkey	CXR • PPD • microbiological staining (Gram, PAS, Ziehl-Neelsen)	Painful mass 39 (85) abscess 11 (24) Axillary LAP 10 (22) Sinus formation 6 (13) Hyperemia and breast tenderness 2 (4)	CNB 46 (100)	Surgical excision 18 (39) Steroids 25 (54) Surgical excision + steroids 3 (6) ABS 19 (41)	35.4 (3–135)	8 (17)
Pandey et al., 2014 [11], observational, prospective cohort study	49	USA =/ Hispanic 39 (80%) African-American 7 = (14%) Other 3 (6%)	CXR • PPD • microbiological smears • Gram stain • cultures for bacteria, fungus, and AFB	Painful breast mass with overlying erythema 39 (80) Painless breast mass 8 (16) Purulent drainage 1 (2) None; image detected 1 (2)	FNA/CNB	Steroids 44 (90) Observation only 3 (6) Surgical excision 2 (4) Final outcome with steroids: Initial response within 2 weeks 44 (100) Complete resolution 35 (80) Lost to follow-up 6 (13) No resolution 3 (7) Resolved with steroids + surgical excision 1 (12) Still receiving steroids 2 (5)	(6–12)	10 (23)
Sheybani et al., 2015 [47], retrospective, monocentric	22	Iran	CXR • PPD • microbiological staining and culture of the affected tissues • PCR for mycobacterium tuberculosis	Painful mass 22 (100) Inflammatory skin changes 15 (68.1) Draining sinus(es) 7 (31.8) Axillary LAP 7 (31.8) Nipple retraction 4 (18.1) Nipple discharge 1 (4.5)	Excision or incision 15 (68.2) CNB 8 (36.3)	Steroids ^1^ 15 (68) Steroids + MTX 6 (27.3) MTX alone 1 (4.5)	11.9 +/−4.4 (6–22).	3 (13.6)
Aghajanzadeh et al., 2015 [4], retrospective, multicentric	206	Iran	Specific stains (Kinyoun AFB, Gomori methenamine silver, and Gram) • microbiological cultures • immunohistochemistry for bacteria and AFB	Mass 181 (88) Axillary LAP 56 (28) Abscess 38 (18) Mass and ulcers 33 (16) Draining sinus 29 (14) Painful mass with inflammation 25 (12) Mass and nipple discharge 25 (12)	Incision + drainage 38 (18) FNA 33 (16) CNB 92 (45) Excision 43 (21)	ABS 206 (100) Successful 6 Unsuccessful 200 Steroids 200 (97) Successful 144 Unsuccessful 56 Steroids + MTX 56 (27) Successful 40 Unsuccessful 16 Steroids + bromocriptine 16 (8) Successful 5 Unsuccessful 11 Surgery + steroids + ABS 11 (5) Successful 11	(9–18)	11 (5)
Freeman et al., 2017 [9], retrospective, monocentric	14	USA/ White 7% Asian 7% Hispanic 36% African American 50%	CXR • Microbiological cultures 71% • Mycobacterial cultures 50% • AFB staining 50%	Mass 14 (100) Pain or tenderness 11 (79) Erythema 7 (50) Swelling 4 (29)	CNB 11 (79) Excision 3 (21%)	ABS alone 1 (7) Steroids alone 0 NSAIDs alone 1 (7) ABS + steroids + MTX 1 (7) Surgery alone 4 (29) Steroids + surgery 1 (7) ABS + surgery 5 (36) ABS + steroids + MTX + surgery 1 (7)	n.r.	n.r.
Prasad et al., 2017 [7], retrospective, monocentric	73	India	Gram staining • bacterial culture • Ziehl-Neelsen staining	Painless mass (61.64) Abscess (38.36)	CNB or Excision	Surgical excision 33 (45) Incision and drainage 26 (36) Expectant approach 13 Steroids 1 (5)	n.r.	12 (16)
Uysal et al., 2017 [24], retrospective, multicentric	720	Turkey	No history of pulmonary TB• no evidence of histologically tuberculous mastitis • negative staining with Ziehl–Neelsen or AFB • negative tissue cultures and CXR findings consistent	Palpable mass 538 (75) Abscess 302 (42) Fistula 215 (42) Axillary LAP 50 (7)	CNB 437 (62) Post-operative pathology 142 (20) Excision 52 (7) Incision 48 (7) FNA 30 (4)	Only medical 258 (36) Surgery then medical 234 (33) Medical then surgery 164 (23) Only surgery 60 (8) **Medical treatment:** ABS 240 (37) Steroids 253 (39) Steroids + ABS 87 (13) Others 57 (9) Antituberculosis therapy 12 (2) MTX 5 (1) **Surgical treatment:** Wide local excision 323 (69) Only abscess drainage 137 (29) Mastectomy 6 (1.3)	16 (8–33)	122 (17)
Azizi et al., 2019 [5], retrospective, multicentric	474	Iran	Staining for fungal and AFB infections	Pain 330 (69.8) Palpable mass 329 (69.4) Inflammation 186 (39.2) Skin lesion 92 (19.4) Nipple retraction 84 (17.7) Nipple discharge 74 (15.6) Arthralgia 22 (4.6)	n.r.	Medical only 173 (36.5) Medical + surgical 163 (34.3) Surgical only 66 (13.9) No treatment (self-limited) 72 (15.1) Medication regimen: ABS 238 (50.2) Steroids 236 (49.7) Immunosuppressants 190 (40.0)	n.r.	118 (24.8)
Yaghan et al., 2020 [49], retrospective, monocentric	44	Jordan	Staining for fungal and AFB infections	Mass 44 (100) Pain 38 (86.4) Abscess-like presentation 16 (36.4) Axillary LAP 13 (29.6) Nipple retraction 3 (6.8) Ulcer, sinus, or fistula formation 3 (6.8)	CNB 42 (95.5) Excision 2 (4.5)	Surgical excision 42 (95.5) Mastectomy 1 (2.3) Steroids ^2^ 19 (43.2)	n.r.	14 (32)
Al Awfi et al., 2023 [6], retrospective, monocentric	64	Oman	Exclusion of TB and fungal infections (exact workflow not reported) positive bacterial culture at the first diagnosis in 5 patients (10.9%)	Mastitis 46 (71.9) Mass 44 (68.8) Abscess 29 (45.3)	CNB 25 (39.1) Excision 39 (60.9)	Medical treatment: ABS 60 (93.8) Steroids 10 (15.6) Surgical treatment: Drainage 46 (73) Excision 24 (38.7) Outcome: Lost to follow up 22 (34.4) Complete resolution 22 (34.4), Partial resolution 10 (15.6), IGM persistence 10 (15.6)	6 months	n.r.

^1^ prednisone, ^2^ prednisolone; Abbreviations: ABS: Antibiotics, AFB: Acid-fast bacilli, CNB: Core needle biopsy, CXR: chest radiography, FNA: Fine needle aspiration, LAP: lymphadenopathy, NSAIDs: non-steroidal anti-inflammatory drugs MTX: Methotrexate, PCR: polymerase chain reaction, PPD: purified protein derivative (Tuberculin skin test), TB: tuberculosis.

**Table 3:** Examples of treatment regimens and response rates in IGM patients treated with steroids.

Study	Number of Patients ^1^	Administrated Regimen	Duration	Resolution Rate n (%)
Erozgen et al., 2010 [63]	25	Prednisolone 16 mg 1-0-1 for 2 weeks, subsequently slow tapering ^2^	2 months	n.r.
Gurleyik et al., 2012 [27]	19	Prednisolone 0.8 mg/kg/day for 1 week, subsequently weekly tapering by 0.2 mg/kg	8 weeks	Partial response ^3^ 19 (100)
Oran et al., 2013 [25]	25	Prednisolone 16 mg 1-0-1 for 2 weeks, subsequently slow tapering for 6 weeks	8 weeks	22 (93)
Pandey et al., 2014 [11]	44	Prednisone 40 mg/day for 2–4 weeks, subsequently tapering by 5–10 mg every 2–4 weeks	up to ≥ 360 days ^4^	35 (80)
Aghajanzadeh et al., 2015 [4]	200	Prednisolone 10–20 mg 1-1-1 subsequently slow tapering along with clinical improvement	2–6 months	144 (72)
Sheybani et al., 2015 [47]	15	Prednisone 0.5–1.0 mg/kg/day for 3–4 weeks, then slow tapering along with clinical improvement	n.r.	10 (66)
Yabanoglu et al., 2015 [62]	44	Prednisolone 0.8 mg/kg/day during the first week and 0.1 mg/kg/day thereafter.	n.r.	n.r.

^1^ patients treated with steroids, ^2^ as a second line therapy after surgical treatment: incision with drainage or total excision, ^3^ secondary surgical excision of residual findings in all patients, ^4^ median 159 days.

**Table 4:** The GRAMAREG study inclusion and exclusion criteria.

Inclusion Criteria	Exclusion Criteria
Histologically confirmed idiopathic granulomatous mastitis by the local pathology (minimally invasive biopsy or histological confirmation on surgical specimen) after 1 January 2015 Female/male patients ≥ 18 years old Signed informed consent form for all patients that are included in the prospective manner (presentation with idiopathic granulomatous mastitis after activation of the study at the study site)	Patients with suspicion of idiopathic granulomatous mastitis but without histological confirmation Suspicion of or confirmed granulomatous mastitis caused by, e.g., duct ectasia, sarcoidosis, mycobacterial, fungal and parasitic infections, foreign materials
